# Human induced pluripotent stem cell-derived hepatic cell lines as a new model for host interaction with hepatitis B virus

**DOI:** 10.1038/srep29358

**Published:** 2016-07-08

**Authors:** Shun Kaneko, Sei Kakinuma, Yasuhiro Asahina, Akihide Kamiya, Masato Miyoshi, Tomoyuki Tsunoda, Sayuri Nitta, Yu Asano, Hiroko Nagata, Satoshi Otani, Fukiko Kawai-Kitahata, Miyako Murakawa, Yasuhiro Itsui, Mina Nakagawa, Seishin Azuma, Hiromitsu Nakauchi, Hironori Nishitsuji, Saneyuki Ujino, Kunitada Shimotohno, Masashi Iwamoto, Koichi Watashi, Takaji Wakita, Mamoru Watanabe

**Affiliations:** 1 Department of Gastroenterology and Hepatology, Tokyo Medical and Dental University, Tokyo, Japan; 2 Department for Liver Disease Control, Tokyo Medical and Dental University, Tokyo, Japan; 3 Institute of Innovative Science and Technology, Tokai University, Isehara, Japan; 4 Division of Stem Cell Therapy, Institute of Medical Science, The University of Tokyo, Tokyo, Japan; 5 Research Center for Hepatitis and Immunology, National Center for Global Health and Medicine, Ichikawa, Japan; 6 Department of Virology II, National Institute of Infectious Diseases, Tokyo, Japan

## Abstract

Hepatitis B virus (HBV) is not eradicated by current antiviral therapies due to persistence of HBV covalently closed circular DNA (cccDNA) in host cells, and thus development of novel culture models for productive HBV infection is urgently needed, which will allow the study of HBV cccDNA eradication. To meet this need, we developed culture models of HBV infection using human induced pluripotent stem cell-derived hepatocyte lineages, including immature proliferating hepatic progenitor-like cell lines (iPS-HPCs) and differentiated hepatocyte-like cells (iPS-Heps). These cells were susceptible to HBV infection, produced HBV particles, and maintained innate immune responses. The infection efficiency of HBV in iPS-HPCs predominantly depended on the expression levels of sodium taurocholate cotransporting polypeptide (NTCP), and was low relative to iPS-Heps: however, long-term culture of iPS-Heps was difficult. To provide a model for HBV persistence, iPS-HPCs overexpressing NTCP were established. The long-term persistence of HBV cccDNA was detected in iPS-HPCs overexpressing NTCP, and depended on the inhibition of the Janus-kinase signaling pathway. In conclusion, this study provides evidence that iPS-derived hepatic cell lines can be utilized for novel HBV culture models with genetic variation to investigate the interactions between HBV and host cells and the development of anti-HBV strategies.

Hepatitis B virus (HBV) infection remains a major public health threat, with more than 240 million humans chronically infected worldwide at risk of developing end-stage liver disease and hepatocellular carcinoma^1^. Nucleos(t)ide analogues suppress HBV replication; however, they cannot eliminate HBV from host cells because of the persistence of HBV covalently closed circular DNA (cccDNA), which serves as the template for viral transcription^2^,^3^. Interferon (IFN)-α is also licensed for chronic hepatitis B treatment; however, its efficacy for HBV clearance is limited^4^. It is essential to elucidate the further mechanisms involved in the persistence of HBV cccDNA in hepatocytes despite the long-term suppression of HBV replication by treatment with nucleos(t)ide analogues. The need for development of novel therapies to eliminate HBV cccDNA is urgent; however, HBV research is hampered by a lack of appropriate *in vitro* infectious models.

Recently, sodium taurocholate cotransporting polypeptide (NTCP) was reported to be an entry receptor for HBV, and overexpression of NTCP in hepatoma cell lines rendered them susceptible to HBV infection^5^. However, hepatoma cell lines lack a number of cellular pathways, including innate immune responses, compared with normal primary hepatocytes^6^,^7^. Of note, IFN-α-related innate immune responses are especially important for HBV elimination from host cells. In contrast to hepatoma cell lines, primary human hepatocytes used as host cells for productive HBV infection are without such problems^8^,^9^. However, the availability of human hepatocytes is limited, because long-term culture is difficult and genetic modification of target genes in these cells is also unavailable. Moreover, the supply of primary human hepatocytes is limited because of donor shortage, and the metabolic functions of such cells are rapidly lost *in vitro*^10^,^11^.

Human induced pluripotent stem (iPS) cells are somatic cells genetically reprogrammed to be pluripotent by the transient expression of genes important for maintaining the properties of embryonic stem cells. Human iPS cells have the potential for differentiation into hepatocyte lineages^12^. Utilization of iPS cells as a model of HBV infection would have several substantial advantages compared with primary hepatocytes and hepatoma cell lines, such as the potential for unlimited expansion and similarity of biological characteristics to normal hepatocytes. Moreover, the influence of the maturity of host cells on the infectivity and life cycle of HBV and specific gene functions in the host’s interactions with HBV can be evaluated using iPS-derived hepatocyte lineages. However, to date, no report has demonstrated the successful HBV infection of iPS-derived hepatic cell lines that have proliferated over the long term.

The aims of this study were to develop appropriate infectious models of HBV using iPS-derived hepatocyte lineages, including immature proliferating hepatic progenitor-like cell lines (iPS-HPCs) and differentiated hepatocyte-like cells (iPS-Heps), and to evaluate the interaction between HBV and such host cells. Here, we demonstrated that human iPS-derived hepatic cells, especially iPS-derived hepatic progenitor-like cell lines overexpressing NTCP (iPS-HPC-NTCP), can be used as a model for HBV persistence, and that these cells maintain innate immune responses against HBV, which is partially lacking in hepatoma cell lines.

## Results

### Expression of an entry receptor for HBV in iPS-HPC lines

**Figure 1:**
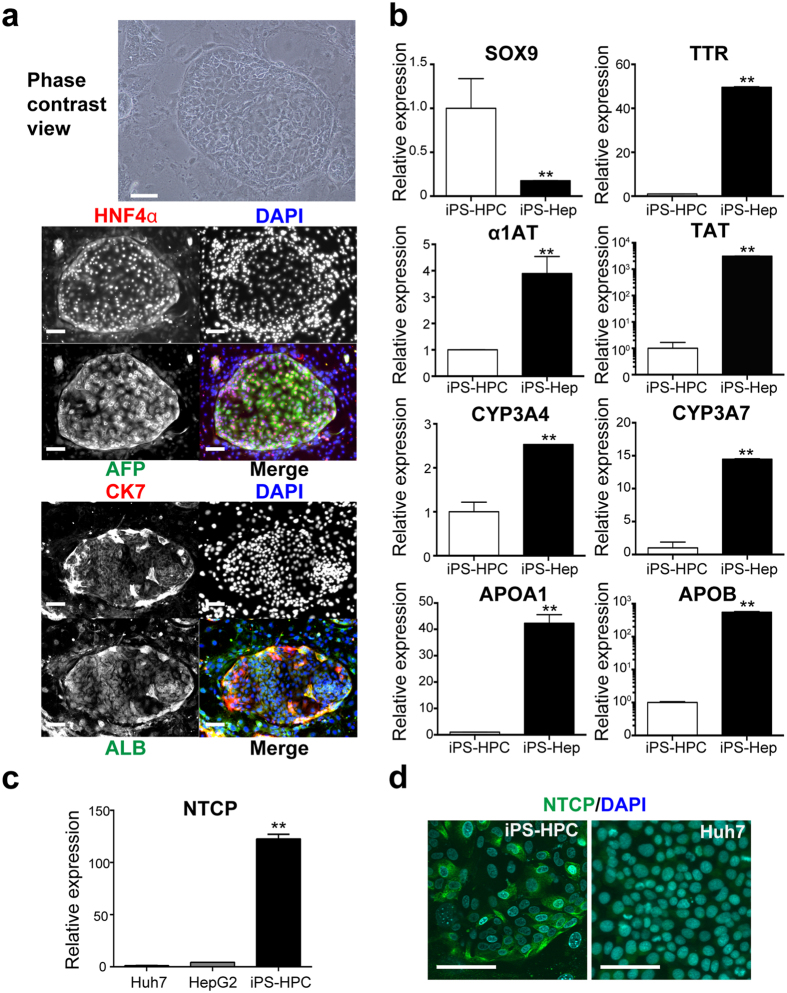
iPS-HPCs express hepatoblast markers and HBV receptor. (**a**) iPS-HPCs formed large colonies on feeder cells. Colonies were immunostained with antibodies against hepatic nuclear factor (HNF) 4α (red), α-fetoprotein (AFP, green), cytokeratin 7(CK7, red), and albumin (ALB, green). Nuclei were counterstained with DAPI (blue). (**b**) Expression of sex determining region Y-box 9 (SOX9), transthyretin (TTR), α1-antitrypsin (α1AT), tyrosine aminotransferase (TAT), cytochrome P450 (CYP) 3A4, CYP3A7, apolipoprotein A-I (APOA1), and apolipoprotein B (APOB) in iPS-HPCs and iPS-Heps. The y-axis represents the ratio of copy numbers in each cell relative to the mean of values in iPS-HPCs. (**c**) Quantitative RT-PCR analysis of NTCP. NTCP expression in Huh7, HepG2, and iPS-HPCs were analyzed. The y-axis represents the ratio of copy numbers in each cell relative to the mean of values in Huh7. (**d**) Representative images of immunostained iPS-HPCs and Huh7. Scale bars: 100 μm; **p < 0.01.

Human iPS cell lines were differentiated into hepatic lineage using 4-step protocol as previously described^12^ with some modifications (sequential addition of activin A, basic fibroblast growth factor + bone morphogenetic protein 4, hepatocyte growth factor, and oncostatin M). Human iPS-HPCs were established from FACS-sorted CD13^+^CD133^+^ cells of the above-mentioned 3rd step (after addition of hepatocyte growth factor), which were cloned onto feeder cells^13^. CD13^**+**^CD133^**+**^ iPS-HPC lines established from iPS cells formed large colonies and were stably cultured on feeder cells for >3 months. These cells expressed the hepatoblast markers, hepatocyte nuclear factor 4α (HNF4α), α-fetoprotein (AFP), cytokeratin-7 (CK7) and albumin (ALB) as previously described (Fig. 1a)^13^. Quantitative RT-PCR analysis showed that the expression of cholangiocyte marker in iPS-HPCs, such as sex determining region Y-box 9, was higher than that in iPS-Heps. On the other hand, the expression of hepatocyte markers in iPS-HPCs, such as transthyretin, α1-antitrypsin, tyrosine aminotransferase, cytochrome P450 (CYP) 3A4, CYP3A7, apolipoprotein A-I, and apolipoprotein B, were lower than those in iPS-Heps (Fig. 1b). Expression of NTCP was >100-fold higher in iPS-HPCs compared with that of hepatoma cell lines (Huh7 and HepG2, Fig. 1c). Immunostaining analysis showed that iPS-HPCs produced NTCP (Fig. 1d).

### HBV infection of iPS-HPCs

**Figure 2:**
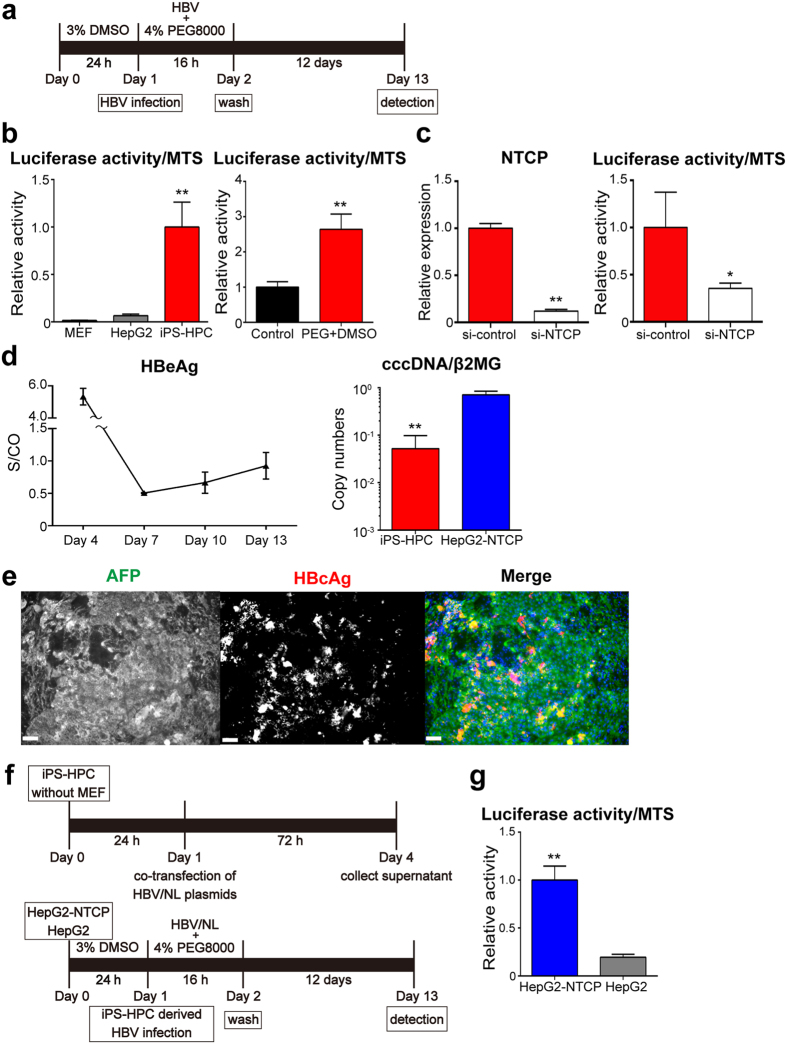
iPS-HPCs are susceptible to HBV infection and produce infectious HBV particles. (**a**) Schema of the schedule for HBV infection in iPS-HPCs. (**b**) MEF, HepG2, and iPS-HPCs were infected with HBV/NL. Levels of relative luciferase activity (normalized MTS assay) are shown as the ratio of the mean relative to iPS-HPCs (left panel). The relative luciferase activity in HBV/NL-infected iPS-HPCs treated without or with polyethylene glycol (PEG) plus dimethyl sulfoxide (DMSO). Control represents iPS-HPCs without PEG plus DMSO (right panel). (**c**) Analysis of knockdown of NTCP in iPS-HPCs. NTCP expression in iPS-HPCs transfected with siRNA against NTCP (si-NTCP) and control siRNA (si-control) were analyzed. The y-axis represents the ratio of copy numbers in each cell relative to the mean of values in si-control group (left panel). The luciferase activity in iPS-HPCs transfected si-control and si-NTCP after HBV/NL infection are shown as the ratio of the mean relative to si-control group (right panel). (**d**) Infection of HepG2.2.15.7-derived HBV to iPS-HPCs. HBeAg in the culture supernatant and cellular cccDNA were detected by CLIA and quantitative PCR analysis, respectively. (**e**) A representative iPS-HPCs infected with HBV immunostained for AFP (green), HBcAg (red), and nuclei (blue). (**f**) Schema of the schedule for HBV infection derived from iPS-HPCs. (**g**) HepG2-NTCP or HepG2 were infected with HBV/NL derived from iPS-HPCs. Expression levels of relative luciferase activity (normalized MTSassay) are shown as the ratio of the mean relative to HepG2-NTCP. Scale bars: 100 μm; *p < 0.05, **p < 0.01.

To determine whether HBV can infect iPS-HPCs, infection by recombinant HBV expressing luciferase, NanoLuc (HBV/NL)^14^ was tested (Fig. 2a). The luciferase activity in iPS-HPCs infected with HBV/NL was significantly higher compared with naïve HepG2 that are nonpermissive for HBV (Fig. 2b, left panel). The luciferase activity in iPS-HPCs was significantly increased by treatment with polyethylene glycol (PEG) plus dimethyl sulfoxide (DMSO) (Fig. 2b, right panel).

Next, to investigate whether the HBV infection in iPS-HPCs depends on NTCP expression, knockdown of NTCP in iPS-HPCs was analyzed. NTCP expression in iPS-HPCs transfected with siRNA against NTCP was significantly decreased relative to that with control siRNA (Fig. 2c, left panel). The luciferase activity in iPS-HPCs transfected with siRNA against NTCP was significantly decreased relative to that with control siRNA after infection of HBV/NL (Fig. 2c, right panel), indicating that the HBV infection in iPS-HPCs depends on NTCP expression.

We investigated the infectivity of HBV derived from HepG2.2.15.7 cells, subcloned from HepG2.2.15 cells, which exhibited the highest HBV replication levels among the established subclones^15^. The concentrations of HBe-antigen (HBeAg) in the culture supernatant of HBV-infected iPS-HPCs gradually increased from day 7 to day 13, and HBV cccDNA was detected in iPS-HPCs at day 13 after infection (Fig. 2d). Quantitative RT-PCR analysis showed that expression levels of the hepatic maturation markers, CYP3A4, CYP3A7, CYP7A1 and ALB were significantly lower in iPS-HPCs than in differentiated iPS-Heps (supplementary Fig. 1). Moreover, immunostaining analysis revealed that HBcore-antigen (HBcAg) was detected in AFP positive iPS-HPCs (Fig. 2e). Taken together, these data indicated that iPS-HPCs at a relatively immature stage of hepatic differentiation can be infected with HBV *in vitro.*

### Production of infectious HBV particles from iPS-HPCs

To assess whether iPS-HPCs can produce infectious particles of HBV, plasmids expressing the HBV genome were transfected into iPS-HPCs. The infectivity of culture supernatants derived from iPS-HPCs transfected with HBV/NL plasmids was tested using HepG2-NTCP and naïve HepG2 (Fig. 2f). iPS-HPCs derived HBV produced luciferase activity in HepG2-NTCP but not naïve HepG2 (Fig. 2g), indicating that iPS-HPCs can produce infectious HBV particles.

### Innate immune responses of iPS-HPCs against HBV

**Figure 3:**
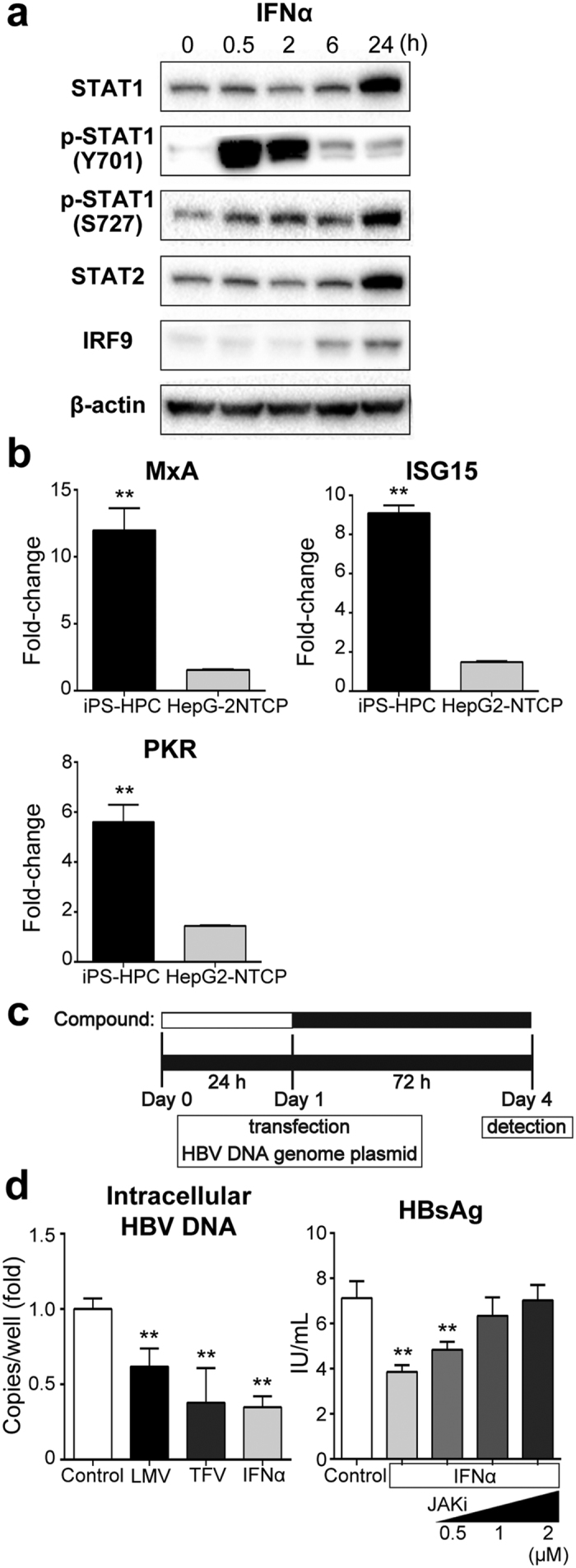
iPS-HPCs respond to IFN stimulation. (**a**) Immunoblot analysis of STAT1, phosphorylated STAT1, STAT2, and IRF9 in iPS-HPCs treated with IFN-α. (**b**) Quantitative RT-PCR analysis of IFN-stimulated genes (MxA, ISG15, and PKR) in iPS-HPCs and HepG2-NTCP treated with or without IFN-α for 48 hours. The y-axis represents the ratio between the copy number of each cell type with IFN-α and the mean of each cell type without IFN-α. (**c**) Schema of the schedule for treatment against iPS-HPCs transfected with plasmids expressing the HBV DNA genome. (**d**) The expression of intracellular HBV DNA and the secretion of HBsAg from iPS-HPCs transfected with plasmids expressing the HBV DNA genome. The effect of IFN-α treatment on the secretion of HBsAg from iPS-HPCs was blocked by the addition of Janus kinase inhibitor I (JAKi) in a dose-dependent manner. **p < 0.01.

To investigate the interaction between host iPS-HPCs and HBV, responses of iPS-HPC to exogenous IFN-α stimulation were analyzed by immunoblot analysis. STAT1 was phosphorylated and increased STAT2 and IRF9 levels were detected in iPS-HPCs treated with IFN-α in a time-dependent manner, indicating that molecules in the IFN-α signaling cascade were activated in iPS-HPCs (Fig. 3a). Moreover, quantitative RT-PCR analysis showed that the expression of IFN-stimulated genes (ISGs), MxA, ISG15, and PKR, in iPS-HPCs treated with IFN-α was significantly increased compared with HepG2-NTCP (Fig. 3b).

To verify the innate immune responses of iPS-HPCs against HBV, the effect of IFN-α on the viral production in iPS-HPCs was investigated. The plasmids expressing 1.2-fold HBV DNA genome, D-IND60, were transfected into iPS-HPCs treated with compounds (Fig. 3c). The copy numbers of HBV DNA in iPS-HPCs treated with lamivudine (LMV), tenofovir (TFV) or IFN-α was significantly decreased compared with non-treat controls (Fig. 3d, left panel). The concentration of HBs-antigen (HBsAg) in the culture supernatant derived from iPS-HPCs treated with IFN-α was also significantly decreased compared with controls. The effect of IFN-α on the reduction of HBsAg concentration was recovered by the addition of Janus-kinase inhibitor I (JAKi) in a dose-dependent manner (Fig. 3d, right panel). These results demonstrated that iPS-HPCs can respond to anti-viral IFN-α stimulation.

### HBV infection of differentiated iPS-Heps

**Figure 4:**
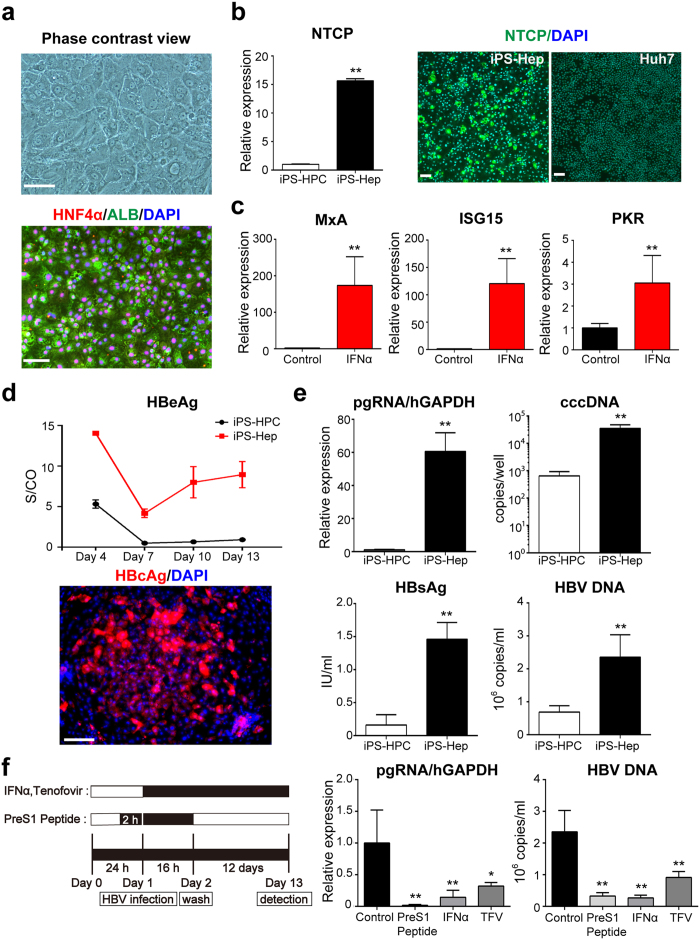
Differentiated iPS-Heps are susceptible to HBV infection. (**a**) Phase contrast view of iPS-Heps. They exhibited morphology similar to hepatocytes, such as clear round nuclei and intercellular spaces (upper panel), and expressed HNF4α (red) and ALB (green, lower panel). Nuclei were counterstained with DAPI (blue). (**b**) The expression of NTCP in iPS-HPCs and iPS-Heps (left panel). The y axis represents the ratio of copy numbers in iPS-Heps relative to the mean of values in iPS-HPCs. Immunostaining of NTCP (green) in iPS-Heps and Huh7 (right panel). (**c**) Quantitative RT-PCR analysis of IFN-stimulated genes (MxA, ISG15, and PKR) in iPS-Heps treated with or without IFN-α for 48 hours. The y-axis represents the ratio between the copy number of cells with IFN-α and the mean of cells without IFN-α. (**d**) Changes of HBeAg concentration in the culture supernatant derived from iPS-Heps and iPS-HPCs infected with HepG2.2.15.7-derived HBV (upper panel). Immunostaining of HBcAg (red) in iPS-Heps infected with HBV (lower panel). Nuclei were counterstained with DAPI (blue). (**e**) Quantification of cellular pregenomic (pg) RNA, cellular cccDNA, HBsAg and HBV DNA in the culture supernatant derived from iPS-HPCs and iPS-Heps. (**f**) Schema of the schedule for treatment of iPS-Heps with drugs against HBV (left panel). Quantification of cellular pgRNA and HBV DNA in the culture supernatant derived from iPS-Heps treated with anti-viral drugs. Scale bars: 100 μm, *p < 0.05, **p < 0.01.

To investigate the efficiency of HBV infectivity in mature host cells, we compared the ability of HBV infection in immature iPS-HPC lines and differentiated iPS-Heps that were cultured by 4-step protocol (after addition of oncostatin M)^12^. Immunostaining analysis showed that iPS-Heps clearly produced both HNF4α and ALB (Fig. 4a). The expression level of NTCP in iPS-Heps was significantly higher than in iPS-HPCs (Fig. 4b). To verify the responses of differentiated iPS-Heps against IFN-α, the expression of IFN-stimulated genes in iPS-Heps were examined by quantitative RT-PCR. Expressions of MxA, ISG15, and PKR in iPS-Heps treated with IFN-α were also significantly increased (Fig. 4c). An infection assay using HBV derived from HepG2.2.15.7 demonstrated that HBeAg concentrations in the culture supernatant derived from iPS-Heps gradually increased and were significantly higher than in iPS-HPCs (Fig. 4d, upper panel). Immunostaining analysis showed that HBcAg production was clearly detected in iPS-Heps (Fig. 4d, lower panel). The expression of pregenomic RNA (pgRNA), copy numbers of HBV cccDNA, concentrations of HBsAg, and HBV DNA copy numbers in the culture supernatant derived from iPS-Heps were significantly increased relative to iPS-HPCs (Fig. 4e). These data indicated that the efficiency of HBV infection in iPS-Heps was higher than in iPS-HPCs.

### Responses of iPS-Heps against anti-viral drugs for HBV

Responses of iPS-Heps against several types of anti-viral drugs for HBV were tested using the HBV infection assay (Fig. 4f, left panel). The expression of pgRNA and the concentration of supernatant HBV DNA in iPS-Heps infected with HBV were significantly decreased in cells treated with PreS1 peptide, IFN-α, or TFV compared with non-treated iPS-Heps, indicating that HBV infection in iPS-Heps was suppressed by these anti-viral drugs (Fig. 4f, right panel).

### NTCP dependency of HBV infection in iPS-HPCs

**Figure 5:**
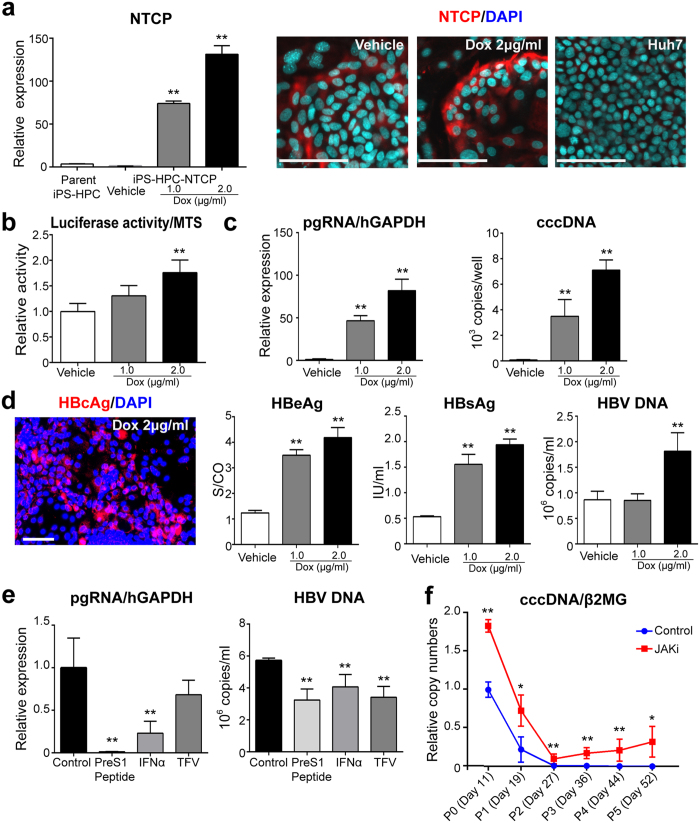
HBV infection in iPS-HPCs is augmented by overexpression of NTCP. (**a**) Quantitative RT-PCR analysis of NTCP (left panel). NTCP expressions in iPS-HPC-NTCP with or without doxycycline (Dox). The y-axis represents the ratio of copy numbers in each cell type relative to the mean of values in parent iPS-HPC lines. Immunostaining of NTCP (red) in iPS-HPC-NTCP with or without Dox and Huh7 (right panel). (**b**) iPS-HPC-NTCP were infected with HBV/NL in the presence or absence of Dox. Levels of relative luciferase activity (normalized MTS assay) are shown as the ratio of the mean relative to iPS-HPC-NTCP without Dox. (**c**) Quantification of cellular pgRNA, cellular cccDNA, HBeAg, HBsAg, and HBV DNA in the culture supernatant derived from iPS-HPC-NTCP infected with HepG2.2.15.7-derived HBV with or without Dox. (**d**) Immunostaining of HBcAg (red) in iPS-HPC-NTCP infected with HepG2.2.15.7-derived HBV. (**e**) iPS-HPC-NTCP were infected with HepG2.2.15.7-derived HBV in the presence of Dox and were treated with anti-viral drugs using the same methods as for Fig. 4f. Intracellular pgRNA and HBV DNA in the culture supernatant derived from iPS-HPC-NTCP were quantified. (**f**) iPS-HPC-NTCP infected with HepG2.2.15.7-derived HBV were cultured with or without JAKi. Infected cells in one well were passaged and divided into 4 wells. Infected cells were passaged 5 times, and were cultured for 52 days. DNA samples were normalized by copy numbers of β2-microglobulin. Control represented iPS-HPC-NTCP infected with HepG2.2.15.7-derived were cultured without JAKi. Scale bars: 100 μm, *p < 0.05, **p < 0.01.

The efficiency of HBV infection in iPS-HPCs was low relative to iPS-Heps (Fig. 4), and depended on the expression level of NTCP (Fig. 2c). To investigate whether the efficiency of HBV infection was enhanced by NTCP expression, we established iPS-HPCs that overexpressed NTCP (iPS-HPC-NTCP). Basal expression of NTCP in iPS-HPC-NTCP without doxycycline (Dox) was similar to the parent iPS-HPCs. Dox treatment dose-dependently increased NTCP expression levels in iPS-HPC-NTCP (Fig. 5a, left panel). Immunostaining showed that Dox treatment increased NTCP protein production (Fig. 5a, right panel). Quantitative RT-PCR analysis demonstrated that hepatic maturation marker expression levels in iPS-HPC-NTCP were unchanged by supplementation of Dox alone (supplementary Fig. 2a). Hepatic maturation of iPS-HPC-NTCP was not inhibited by NTCP overexpression or Dox treatment (supplementary Fig. 2b).

Infection assay of HBV/NL revealed that luciferase activity in iPS-HPC-NTCP was dose-dependently increased by Dox (Fig. 5b). An infection assay using HepG2.2.15.7-derived HBV showed that pgRNA expression, HBV cccDNA copy numbers, concentrations of HBeAg, HBsAg, and HBV DNA copy numbers in the culture supernatant derived from iPS-HPC-NTCP were significantly and dose-dependently increased by Dox (Fig. 5c). Immunostaining showed HBcAg production in iPS-HPC-NTCP (Fig. 5d). Thus, the expression level of NTCP is an essential factor for the efficiency of HBV infection in iPS-HPCs.

The responses of iPS-HPC-NTCP to several types of anti-viral drugs for HBV were tested by an HBV-infection assay. pgRNA expression was significantly decreased in iPS-HPC-NTCP treated with PreS1 peptide or IFN-α (Fig. 5e, left panel). Supernatant HBV DNA copy numbers were significantly decreased in iPS-HPC-NTCP treated with PreS1 peptide, IFN-α, or TFV (Fig. 5e, right panel).

### Long-term persistence of HBV cccDNA in host iPS-HPC lines

To determine whether HBV cccDNA showed long-term persistence, iPS-HPC-NTCP were infected with HBV in the presence or absence of JAKi and passaged repeatedly on feeder cells for 52 days, after which HBV cccDNA was analyzed. The expression of ISGs in iPS-HPC-NTCP was induced by IFN-α as well as parent iPS-HPCs (supplementary Fig. 3). Copy numbers of cccDNA decreased during cell division by segregation of cccDNA^16^. After 5 passages of iPS-HPC-NTCP (52 days of culture), HBV cccDNA was detected in iPS-HPC-NTCP treated with vehicle alone and JAKi, and cccDNA copy numbers per cell were significantly higher in cells with JAKi relative to vehicle alone (Fig. 5f). These results demonstrated that HBV cccDNA persisted in iPS-HPC-NTCP for a long term, and suggested that suppression of JAK-mediated innate immune response is associated with persistence of HBV cccDNA *in vitro.*

## Discussion

This study provides the first evidence that iPS-HPC lines can be utilized for an experimental model of HBV infection and virus-host interactions. iPS-HPCs, purified as a CD13^+^133^+^ population from hepatoblast-like cells derived from iPS cells, were stably cultured for a long period and maintained hepatic progenitor phenotypes (Fig. 1). These cells were susceptible to HBV infection and produced HBV infectious particles (Fig. 2). Moreover, iPS-HPCs responded to IFN-α stimulation, and the induction of ISGs was significantly higher compared with hepatoma cell lines (Fig. 3). One advantage of iPS-HPCs as host cells for HBV is their ability to maintain a stable hepatic cell phenotype during long-term culture in contrast to iPS-Heps. In addition, FACS sorting allows the isolation of a homogenous population of iPS-HPCs, whereas iPS-Heps are a relatively heterogeneous population containing contaminating cells such as mesenchymal cells that spontaneously differentiate from iPS cells. A previous report showed that iPS-derived hepatocyte lineages were susceptible to HBV when they differentiated from hepatoblast-like cells to hepatocyte-like cells^17^. Our data indicated that the efficiency of HBV infection in iPS-HPCs was significantly lower compared with iPS-Heps (Fig. 4) as previously reported^17^. However, in contrast to that report, our data clearly showed that iPS-HPCs in the hepatoblast-stage exhibited HBV infectivity. The discrepancy might be explained by the stable phenotype of our iPS-HPC lines and high sensitivity infection assay using HBV/NL.

To overcome the low efficiency of HBV infection in iPS-HPCs, we focused on NTCP expression. A previous report indicated NTCP is a key receptor for HBV infection because hepatoma cells that lacked HBV infectivity were infected with HBV when they overexpressed NTCP. The expression levels of NTCP were approximately 1 × 10^4^ and 1 × 10^3^ times higher in primary hepatocytes and differentiated HepaRG, respectively, relative to hepatoma cells such as Huh7 or HepG2^5^. The expression levels of NTCP in HepG2-NTCP were also approximately 1 × 10^4^ times higher than HepG2^18^. The expression levels of NTCP in iPS-HPCs, iPS-Heps, and iPS-HPC-NTCP were approximately 1 × 10^2^, 1 × 10^3^, and 1 × 10^4^ times higher relative to hepatoma cells, respectively (Figs 1c, 4b and 5a). These data suggest HBV infectivity is predominantly correlated with NTCP expression *in vitro*. However, the infection efficiency of iPS-HPC-NTCP was not higher than iPS-Hep (Fig. 5c,d), suggesting HBV infection might also depend on another factor/receptor, such as the asialoglycoprotein receptor (ASGPR), a candidate HBV receptor^19^,^20^. Our data showed that ASGPR expression in iPS-Hep was significantly increased compared with iPS-HPC (supplementary Fig. 4); however, HBV infection efficiency in iPS-HPCs overexpressing ASGPR was similar to control iPS-HPCs (data not shown). Taken together, our data strongly suggested that NTCP expression was the most important factor for host cell HBV infectivity *in vitro*.

Innate immune responses are important for the eradication of HBV^21^,^22^. NTCP overexpression in hepatoma cell lines renders them susceptible to HBV infection; however, hepatoma cells are defective for many cellular pathways associated with type 1 interferon related innate immune response^6^,^7^. Our data (Figs 3 and 4c, and supplementary Fig. 3) and those of others^23^ showed the innate immune system was intact in hepatic cells derived from human pluripotent stem cells. Therefore, a cell bank containing iPS cell lines with wide genetic variation could help elucidate the virus-host interaction in chronic HBV infection and aid drug development for HBV infection.

Human primary hepatocytes are available for modeling HBV infection *in vitro*. However, it is impossible to use primary hepatocytes for large-scale drug screening because of the donor shortage and their lack of proliferation *in vitro*. Human iPS cell lines could be established from donors with genetic variations, and our strategy for the HBV infection model requires no human liver sample. Therefore, iPS-HPC and iPS-HPC-NTCP could be utilized for drug screening, studying virus-host interactions, and genetic modification in host cells in the future.

We demonstrated that immature iPS-HPC lines supported the HBV life cycle although HBV is generally considered to infect only mature hepatocytes. Currently it is unknown why HBV cccDNA persist in mature hepatocytes even though they are constantly proliferating, and HBV replication can be inhibited long-term by nucleos(t)ide analogues. Upon injurious conditions, unique populations of epithelial cells with an immature phenotype, termed liver progenitor cells, emerge and expand, and may contribute to tissue regeneration^24^. Our data suggest that HBV may persist in hepatic progenitor-like cells over long periods. Further study regarding the association between hepatic cell differentiation and HBV life cycle is required to understand the mechanism of persistent infection.

In conclusion, human iPS-derived hepatic cells, especially iPS-HPC-NTCP, support the HBV life cycle and can be used as a model for HBV infection. These cells maintain innate immune responses against HBV compared with hepatoma cell lines. This study provides the first evidence that iPS-HPCs can be utilized as an experimental model to investigate HBV-host interactions, develop novel therapies, and determine the association between HBV persistence and cell differentiation.

## Materials and Methods

### Cell Culture

A human iPS cell line, RIKEN 2F (a daughter cell line of RCB0197 HUC-Fm that was established from Japanese male umbilical cord fibroblasts reprogrammed by the overexpression of Oct3/4, Sox2, Klf4, and c-Myc transduced by retroviral vectors) was cultured as described previously^25^. RIKEN 2F cell line was differentiated into the hepatic lineage using a 4-step protocol as previously described^12^ with some modifications. Briefly, semi-confluent human iPS cells were cultured in RPMI 1640 (Sigma, St. Louis, MO, USA) containing 2% B27 supplement (Thermo Fisher Scientific, Waltham, MA, USA). Cells were cultured with 100 ng/mL recombinant human activin A (PeproTech, Rocky, NJ, USA) on days 0–4, 10 ng/mL basic fibroblast growth factor (Wako Pure Chemicals, Osaka, Japan) and 20 ng/mL recombinant human bone morphogenetic protein 4 (PeproTech) on days 5–8, and 40 ng/mL recombinant human hepatocyte growth factor (PeproTech) on days 9–12. Then they were cultured in a hepatocyte culture media bullet kit (Lonza, Basel, Switzerland) and stimulated with 20 ng/mL recombinant human oncostatin M (R&D Systems, Minneapolis, MN, USA) on days 13–16. Cells cultured by the 4-step protocol were termed iPS-derived hepatocyte-like cells (iPS-Heps).

CD13^**+**^CD133^**+**^ cells prepared according to the above protocol on day 12 were sorted by FACS and were cloned onto feeder cells, which were cultured and passaged as iPS-derived hepatic progenitor-like cells (iPS-HPCs)^13^,^26^. iPS-HPCs were cultured as previously described^13^. HepG2 overexpressing NTCP (HepG2-NTCP [C4])^27^, HepG2, and Huh7 were used as control cells.

### Establishment of iPS-HPC lines overexpressing NTCP

We constructed a self-contained, drug-inducible expression vector based on the PiggyBac transposon^28^. This vector constitutively expresses a neomycin resistance gene and an rtTA transactivator element, which mediates Dox-dependent activation of cDNA cassettes controlled by the tetO promoter. Activation of gene expression in response to Dox may be indirectly monitored by co-incident green fluorescent protein activation. Using the Gateway cloning technique, we produced a derivative vector expressing human NTCP. The Tet-NTCP vector was transfected together with PiggyBac transposase into the RIKEN-2F iPS cell line and was selected using media containing G418 for 3 days to generate pooled 2F iPS^NTCP^ cells containing genomic transposon integrations. The resultant 2F iPS^NTCP^ cells were differentiated into the hepatic lineage as described above, and the CD13^**+**^CD133^**+**^ fraction derived from NTCP-iPS cells was sorted and cloned onto mouse embryonic fibroblast feeder cells, which were used as iPS-HPC-NTCP host cells.

### Preparation and infection of HBV

HBV used in the infection study was mainly derived from the culture supernatant of HepG2.2.15.7 cells, subcloned from HepG2.2.15 cells, which exhibited the highest HBV replication levels among the established subclones^15^. HBV was prepared and infected as described^29^. Host cells were infected with HBV at 1.8 × 10^4^ genome equivalent/cell. Cells were treated with 3% DMSO (Thermo Fisher Scientific) before infection and infected in the presence of 4% PEG 8000 (Promega, Madison, WI, USA) at 37 °C for 16 h as previously described^27^,^30^. For inhibition assays using PreS1 peptide, 100 nM PreS1 peptide was added into the medium 2 hours before infection^29^,^31^. At the time of HBV infection, 1,000 IU/ml IFN-α and 1 μM TFV were concomitantly added into the medium.

### Infection assay using HBV expressing reporter gene

A recombinant HBV/NL was produced by cotransfecting a plasmid containing a 1.2-fold HBV genome carrying the NanoLuc gene with a plasmid bearing a packaging-defective 1.2-fold HBV genome as previously described^14^. Infection was performed as described above. Activity of NanoLuc was quantified using the NanoGlo luciferase assay system (Promega) and GloMax discover system (Promega). Cell viability was quantified using the MTS assay kit (Promega).

### Knockdown of NTCP

Human NTCP-targeted or control siRNAs (Thermo Fisher Scientific) were transfected into the cells at a final concentration of 30 nM using Lipofectamine RNAiMAX (Thermo Fisher Scientific) according to the manufacturer’s protocol, and were cultured for 72 hours. Then, the cells were infected with HBV/NL, and activity of NanoLuc and cell viability were quantified as described above.

### Assay for innate immune response against HBV production

Replication of HBV can be mimicked by transfection of plasmids expressing the HBV genome into hepatic host cells^32^. Briefly, iPS-HPCs were enriched from feeder mouse embryonic fibroblasts using feeder removal microbeads (Miltenyi Biotec, Bergisch Gladbach, Germany) and a magnetic cell sorter (Miltenyi Biotec), and seeded onto collagen-coated dishes. Host iPS-HPCs were transfected with 1 μg plasmids expressing the HBV genome, D-IND60, in 100 μL Opti-MEM (Thermo Fisher Scientific) using ViaFect transfection reagent complex (Promega), and were cultured for 3 days. Then, 1 μM LMV, 1 μM TFV, and 1,000 IU/ml IFN-α were added to the medium for drug inhibition assays. For IFN-α inhibition assays, 1,000 IU/ml IFN-α with or without 0.5–2.0 μM JAKi (Pyridone 6) was added to the medium.

### Assay for production of infectious HBV particles

For the transfection of plasmids expressing the HBV genome, iPS-HPCs were enriched and seeded as described above. Host iPS-HPCs were transfected with HBV/NL plasmids, and culture supernatants were collected at 72 hours after transfection. HBV/NL was enriched from culture supernatants using precipitation with 10% PEG8000 and 2.3% NaCl, and the concentrated viral solution was infected in HepG2-NTCP or HepG2.

### Statistics

GraphPad Prism software (GraphPad Software, San Diego, CA, USA) was used to calculate the standard deviation (SD) and statistical significance between samples (two-tailed Student’s *t* test); p values < 0.05 were considered statistically significant. In all graphs, bars represent the mean ± SD of three or four separate experiments.

## Additional Information

**How to cite this article**: Kaneko, S. *et al*. Human induced pluripotent stem cell-derived hepatic cell lines as a new model for host interaction with hepatitis B virus. *Sci. Rep.* **6**, 29358; doi: 10.1038/srep29358 (2016).

## Supplementary Material

Supplementary Information
