# Supplementary material for: Human induced pluripotent stem cell-derived hepatic cell lines as a new model for host interaction with hepatitis B virus
Source: Sci Rep. 2016 Jul 8;6:29358. doi: 10.1038/srep29358 (PMC4937433; doi:10.1038/srep29358)
Supplement: Supplementary Information [file srep29358-s1.pdf]

Supplementary Information

Human induced pluripotent stem cell-derived hepatic cell lines as a new model for host interaction with hepatitis B virus

Shun Kaneko[1,a], Sei Kakinuma[1,2,a], Yasuhiro Asahina[1,2,*], Akihide Kamiya[3], Masato Miyoshi[1], Tomoyuki Tsunoda[1], Sayuri Nitta[1], Yu Asano[1], Hiroko Nagata[1], Satoshi Otani[1], Fukiko Kawai-Kitahata[1], Miyako Murakawa[1], Yasuhiro Itsui[1], Mina Nakagawa[1], Seishin Azuma[1], Hiromitsu Nakauchi[4], Hironori Nishitsuji[5], Saneyuki Ujino[5], Kunitada Shimotohno[5], Masashi Iwamoto[6], Koichi Watashi[6], Takaji Wakita[6], Mamoru Watanabe[1,*]

1 Department of Gastroenterology and Hepatology, Tokyo Medical and Dental University, Tokyo, Japan

2 Department for Liver Disease Control, Tokyo Medical and Dental University, Tokyo, Japan

3 Institute of Innovative Science and Technology, Tokai University, Isehara, Japan

4 Division of Stem Cell Therapy, Institute of Medical Science, The University of Tokyo, Tokyo, Japan

5 Research Center for Hepatitis and Immunology, National Center for Global Health and Medicine, Ichikawa, Japan

6 Department of Virology II, National Institute of Infectious Diseases, Tokyo, Japan

Supplementary Materials and Methods

Materials

Reagents and suppliers were: Dulbecco’s modified Eagle’s medium (DMEM), dexamethasone (Dex), 4',6-diamidine-2'-phenylindole dihydrochloride (DAPI), 0.05% trypsin-EDTA, and lamivudine (Sigma, St. Louis, MO, USA); fetal calf serum (FCS), (Bio West, Nuaillé, France); DMEM/F-12, DMEM/F-12 GlutaMAX™, 100× insulin/transferrin/selenium (ITS), 200 mM L-Glutamine, 100× non-essential amino acid, Knockout Serum Replacement™, 2-mercaptoethanol, and 2.5% trypsin solution, (Thermo Fisher Scientific, Waltham, MA, USA); penicillin-streptomycin mixed solution, 1 M HEPES Buffer Solution, 100× antibiotic‑antimycotic mixed stock solution, and G418 disulfate aqueous solution (Nakalai Tesque, Kyoto, Japan); Engelbreth-Holm-Swarm (EHS) gel (Corning International, Corning, NY, USA); S-medium, TC-Protector (DS Pharma Biomedical, Osaka, Japan); Y27632, CHIR99021, nicotinamide (Wako Pure Chemicals, Osaka, Japan); A83-01 (Tocris Bioscience, Bristol, UK); recombinant human epidermal growth factor (EGF) (PeproTech, Rocky Hill, NJ, USA); doxycycline (Dox) (Clonetech, Mountain View, CA, USA); mitomycin C (Kyowa Hakko Kirin, Tokyo, Japan); interferon (IFN)-α (Merck, Kenilworth, NJ, USA); Janus-kinase Inhibitor I (Pyridone 6) (Merck Millipore, Darmstadt, Germany); and tenofovir (Selleckchem, Houston, TX, USA).

Quantitative reverse-transcriptase polymerase chain reaction (RT-PCR) analysis

Real-time PCR analyses of quantification of hepatitis B virus (HBV) DNA and HBV covalently closed circular (ccc) DNA were performed as previously described[1]. Total RNA from cultured cells was isolated using RNeasy Mini Kit (Qiagen, Hilden, Germany), and viral RNA was isolated using the RNeasy Mini Kit and RNase-Free DNase Set (Qiagen). First-strand cDNA was synthesized from 1 μg of RNA sample using PrimescriptII reverse transcriptase (Takara Bio, Shiga, Japan) according to the manufacturer's instructions. The resultant first cDNA samples were normalized by the number of GAPDH copies quantified by TaqMan PCR. The mRNA expression levels for HBV-RNA[2] and interferon-stimulated genes (MxA, ISG15, and PKR)[3] were quantified using QuantiTect SYBR Green PCR Kit (Qiagen). The mRNA expression levels for other genes were quantified using a Universal Probe Library (Roche Diagnostics, Indianapolis, IN, USA). Real-time PCR was performed using a StepOnePlus Real Time PCR system (Thermo Fisher Scientific). Primer sequences are listed in Supplementary Table 1.

Quantification of HBs and HBe antigens (Ags)

The concentrations of HBsAg and HBeAg in culture supernatants were quantified by a Chemiluminescent immunoassay, Architect HBsAg reagent kit and Architect HBeAg reagent kit (Abbott Japan, Tokyo, Japan), respectively, according to the

manufacturer's instructions.

Immunostaining of cultured cells

Cultured cells were fixed with 4% paraformaldehyde/PBS or 50% acetone/50% methanol and permeabilized using 0.5% Triton X-100/PBS. After blocking with 10% Blocking One (Nakalai, Tokyo, Japan)/PBS, cells were incubated in primary antibodies overnight at 4°C. The cells were washed with PBS three times and were incubated with diluted secondary antibodies for 2 hours at room temperature. Then, the cells were washed with PBS and their nuclei were stained with DAPI. For each analysis, addition of an appropriate immune serum was used as a negative control. The microscopic photographs were taken using a fluorescent microscope (BZ-X710, Keyence, Osaka, Japan) or confocal laser microscope (FV10i, Olympus, Tokyo, Japan). The primary and second antibodies are listed in Supplementary Table 2.

Immunoblot analysis

The protocol for immunoblotting was previously described[4]. Briefly, tissue samples were homogenized in RIPA buffer (50 mM, pH 8.0, Tris-HCl, 150 mM NaCl, 0.1% NP-40, 1% sodium deoxycholate, 1% sodium dodecyl sulfate) containing a Halt proteinase and phosphatase inhibitor cocktail (Thermo Fisher Scientific). After sodium dodecyl sulfate/polyacrylamide gel electrophoresis of cell homogenates (20 µg protein)

and transfer to PVDF membranes, blots were incubated with primary antibodies (Supplementary Table 2), followed by incubation with peroxidase-labeled secondary antibodies (GE Healthcare, Uppsala, Sweden) and visualization using the ECL Western Blotting Analysis System (GE Healthcare).

Supplementary Table 1. List of primers for quantitative RT-PCR

<Hepatocyte marker>

AFP	F	5’- ATGGCCATCACCAGAAAAAT -3’	ALB	F	5’- AATGTTGCCAAGCTGCTGA -3’
	R	5’- CATAAGTGTCCGATAATAATGTCAGC -3’		R	5’- CTTCCCTTCATCCCGAAGTT -3’
	Probe	Universal ProbeLibrary Probes #66		Probe	Universal ProbeLibrary Probes #27
APOA1	F	5’- CCTTGGGAAAACAGCTAAACC -3’	APOB	F	5’- GACGACTTTTCTAAATGGAACTTCTAC -3’
	R	5’- CCAGAACTCCTGGGTCACA -3’		R	5’- CTCAGTTTTGAATATGGTGAGTTTTT -3’
	Probe	Universal ProbeLibrary Probes #39		Probe	Universal ProbeLibrary Probes #55
ASGPR	F	5’- GCTGACGCCGACAACTACT -3’	α1AT	F	5’- CAGAGCTTGAGGAGAGCTTGA -3’
	R	5’- CCTATGTGGTGCTGGACAAAT -3’		R	5’- AAAATGCCTGATGCTGAAGAC -3’
	Probe	Universal ProbeLibrary Probes #19		Probe	Universal ProbeLibrary Probes #15
CYP3A4	F	5’- AAGAAACTGAGTCCCACAAAGC -3’	CYP3A7	F	5’- CAATGGGATGTTTATTCCCAAA -3’
	R	5’- CCAGCAAAAATAAAGATAATTGATTG -3’		R	5’- TGTCCTTGTTCTTTTTACTGAACCT -3’
	Probe	Universal ProbeLibrary Probes #50		Probe	Universal ProbeLibrary Probes #77
CYP7A1	F	5’- GCAGGCACCTGTAGTCTTAGC -3’	GAPDH	F	5’- AGCCACATCGCTCAGACAC -3’
	R	5’- CGGAGACGGGATCTCACTA -3’		R	5’- GCCCAATACGACCAAATCC -3’
	Probe	Universal ProbeLibrary Probes #64		Probe	Universal ProbeLibrary Probes #60
NTCP	F	5’- CATGCGCTATGTCATCAAGG -3’	SOX9	F	5’- AACGCCTTCATGGTGTGG -3’
	R	5’- TGATGCTCTTCCCCACATT -3’		R	5’- TCTCGCTCTCGTTCAGAAGTC -3’
	Probe	Universal ProbeLibrary Probes #53		Probe	Universal ProbeLibrary Probes #4
TAT	F	5’- TGAAGTTACCCAGGCAATGA-3’	TTR	F	5’- GCCGTGCATGTGTTCAGA -3’
	R	5’- TCCCGACTGGATAGGAAGC -3’		R	5’- GCTCTCCAGACTCACTGGTTTT -3’
	Probe	Universal ProbeLibrary Probes #37		Probe	Universal ProbeLibrary Probes #66

AFP, α-fetoprotein; ALB, albumin; APOA1, apolipoprotein A-I; APOB, apolipoprotein B; ASGPR, asialoglycoprotein receptor; α1AT, α1-antitrypsin; CYP3A4, cytochrome P450, family 3, subfamily A, polypeptide 4; CYP3A7, cytochrome P450, family 3, subfamily A, polypeptide 7; CYP7A1, cytochrome P450, family 7, subfamily A, polypeptide 1; GAPDH, glyceraldehyde 3-phosphate dehydrogenase; NTCP, *sodium* taurocholate cotransporting polypeptide; SOX9, sex determining region Y-box 9; TAT,

tyrosine aminotransferase; TTR, transthyretin; F, forward primer; R, reverse primer.

<HBV and Interferon stimulated genes >

Genotype D HBV DNA	F	5’-ACTCACCAACCTCCTGTCCT-3’	Genotype D cccDNA	F	5’-CGTCTGTGCCTTCTCATCTGC-3’
	R	5’-GACAAACGGGCAACATACCT-3’		R	5’-GCACAGCTTGGAGGCTTGAA-3’
	Probe	5’-TATCGCTGGATGTGTCTGCGGCGT-3’		Probe	5’-CTGTAGGCATAAATTGGT-3’(MGB)
Genotype D HBV 3.5 kb transcripts	F	5’-GAGTGTGGATTCGCACTCC-3’	Genotype D HBV specific transcripts	F	5'-TCACCAGCACCATGCAAC-3'
	R	5’-GAGGCGAGGGAGTTCTTCT-3’		R	5'- AAGCCACCCAAGGCACAG-3'
HBV (HBV/NL)	F	5'-CCTCTGCCTAATCATCTCATGTTC-3'	NL (HBV/NL)	F	5’- ATGGTCTTCACACTCGAAG-3’
	R	5'-CGGTGTCGAGGAGATCTCGAATAG-3'		R	5’- TCAGGACAATCCTTTGGATC -3’
MxA	F	5'-GCCAGCAGCTTCAGAAGGCCATGCTGCAGC-3'	ISG15	F	5'-CACCTGAAGCAGCAAGTGAGCGGGCTGGAG-3'
	R	5'-GGGCAAGCCGGCGCCGAGCCTGCGTCAGCC-3'		R	5'-CCGCAGGCGCAGATTCATGAACACGGTGCT-3'
PKR	F	5'-TTTGAAACATCAAAGTTTTTCACAGACCTA-3'	β2-MG	F	5'-ATGTGTCTGGGTTTCATCCATCCG-3'
	R	5'-CACAGTCAAGGTCCTTAGTATTTCAGATGT-3'		R	5'-CCCACTTAACTATCTTGGGCTGTG-3

HBV, hepatitis B virus; NL, NanoLuc; MxA, myxovirus resistance gene A; PKR, protein kinase R; ISG15, interferon stimulated gene 15; β2-MG, β2-microglobulin; IFN, interferon; F, forward primer; R, reverse primer.

Supplementary Table 2. List of antibodies for immunostaining and immunoblotting

Primary antibodies for immunostaining	Dilution	Source	Catalog number
HNF4α	1/500	Santa Cruz	sc-6556
α-fetoprotein	1/300	Spring	E-2954
Albumin	1/1000	DAKO	A0001
Cytokeratin-7 (CK7, OV-TL 12/30)	1/1000	DAKO	M7018
NTCP (SLC10A)	1/500	Abcam	ab131084
Mouse monoclonal antibody Hepatitis B virus (core antigen)	1/100	Leica Biosystems	NCL-HBcAg-506
Mouse monoclonal antibody Hepatitis B virus (surface antigen)	1/100	Leica Biosystems	NCL-HBsAg-2

Secondary antibodies for immunostaining	Dilution	Source	Catalog number
AlexaFluor-488 donkey anti-rabbit IgG	1/1000	Thermo Fisher	A21206
AlexaFluor-555 donkey anti-rabbit IgG	1/1000	Thermo Fisher	A31572
AlexaFluor-546 goat anti-mouse IgG	1/1000	Thermo Fisher	A11003
AlexaFluor-568 donkey anti-goat IgG	1/1000	Thermo Fisher	A11057

Primary antibodies for immunoblots	Dilution	Source	Catalog number
STAT1 (p84/p91)	1/1000	Santa Cruz	sc-464
STAT2 (A-7)	1/1000	Santa Cruz	sc-1668
Phospho-Stat1 (Ser727)	1/1000	Cell Signaling	9177
Phospho-Stat1 (Tyr701, D4A7)	1/1000	Cell Signaling	7649
ISGF3p48 (IRF9, H-143)	1/1000	Santa Cruz	sc-10793
β-actin	1/5000	Sigma	A5441

HNF4α, hepatocyte nuclear factor 4α; NTCP, sodium taurocholate cotransporting polypeptide; STAT, signal transduction and activator of transcription; ISGF, interferon-stimulated-gene factor; IRF, interferon-regulatory factor.

Supplementary Figures

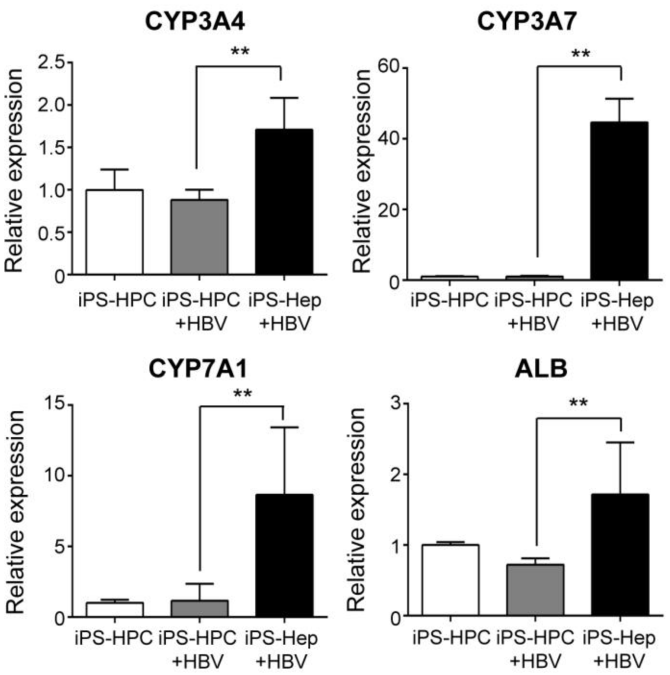

Supplementary Figure 1. iPS-HPCs are infected with HBV at the immature stage of hepatic differentiation in vitro. Quantitative RT-PCR analysis of CYP3A4, CYP3A7, CYP7A1, and ALB. Expressions in iPS-HPCs without or with HBV infection and in iPS-Heps with HBV infection were analyzed. The y-axis represents the ratio of copy numbers relative to the mean of values in iPS-HPCs without HBV infection. **$p < 0.01$.

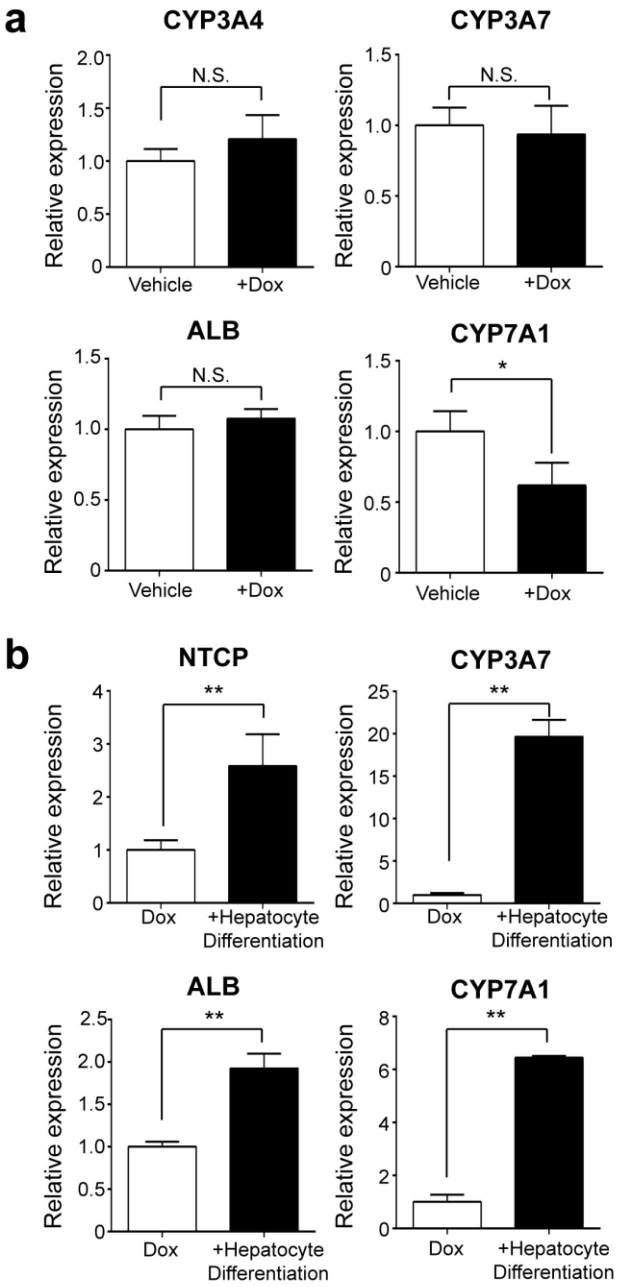

Supplementary Figure 2. Hepatocyte maturation of iPS-HPC-NTCP is not affected by Dox. (a) Quantitative RT-PCR analysis of CYP3A4, CYP3A7, ALB, and CYP7A1. Expressions in iPS-HPC-NTCP with or without 2 μg/mL Dox were analyzed. The y-axis represents the ratio of copy numbers relative to the mean of values in iPS-HPC-NTCP without Dox. (b) Quantitative RT-PCR analysis of NTCP, CYP3A7, ALB, and CYP7A1. iPS-HPC-NTCP were cultured in medium for hepatocyte differentiation (Hepatocyte culture media bullet kit supplemented with 20 ng/ml recombinant human oncostatin M) with 2 μg/mL Dox or iPS-HPC medium with 2 μg/mL Dox. The y-axis represents the ratio of copy numbers relative to the mean of values for cells in iPS-HPC medium with 2 μg/mL Dox. $^*p < 0.05$, $^{**}p < 0.01$.

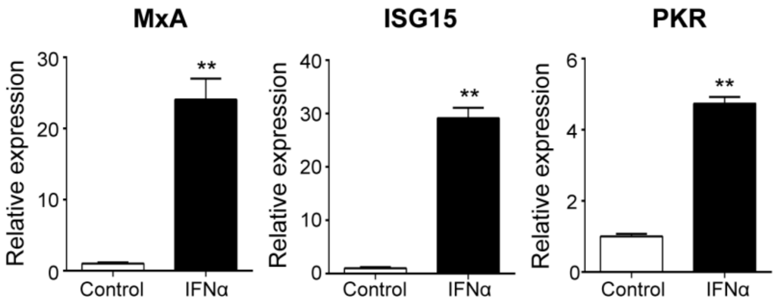

Supplementary Figure 3. Innate immune responses are induced in iPS-HPC-NTCP. Quantitative RT-PCR analysis of IFN-stimulated genes (MxA, ISG15, and PKR) in iPS-HPC-NTCP treated with or without IFN-α for 48 hours. The y-axis represents the ratio between the copy number of cells with IFN-α and the mean of cells without IFN-α. **p < 0.01.

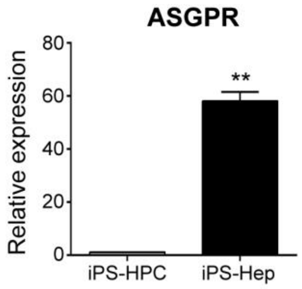

Supplementary Figure 4. Asialoglycoprotein receptor (ASGPR) expression in iPS-HPCs and iPS-Heps. The y-axis represents the ratio of copy number relative to the mean of values in iPS-HPCs. **$p < 0.01$.

References

1. Watashi, K. *et al.* Interleukin-1 and tumor necrosis factor-alpha trigger restriction of hepatitis B virus infection via a cytidine deaminase activation-induced cytidine deaminase (AID). *J. Biol. Chem.* **288**, 31715-31727 (2013).
2. Yan, H. *et al.* Sodium taurocholate cotransporting polypeptide is a functional receptor for human hepatitis B and D virus. *Elife* **1**, e00049 (2012).
3. Itsui, Y. *et al.* Expressional screening of interferon-stimulated genes for antiviral activity against hepatitis C virus replication. *J. Viral. Hepat.* **13**, 690-700 (2006).
4. Kiyohashi, K. *et al.* Wnt5a signaling mediates biliary differentiation of fetal hepatic stem/progenitor cells in mice. *Hepatology* **57**, 2502-2513 (2013).
